# Prevalence and Correlates of Food and/or Housing Instability among Men and Women Post-9/11 US Veterans

**DOI:** 10.3390/ijerph21030356

**Published:** 2024-03-18

**Authors:** Yasmin S. Cypel, Shira Maguen, Paul A. Bernhard, William J. Culpepper, Aaron I. Schneiderman

**Affiliations:** 1 Health Outcomes Military Exposures, Epidemiology Program, Office of Patient Care Services, US Department of Veterans Affairs, Washington, DC 20420, USA; paul.bernhard@va.gov (P.A.B.); william.culpepper@va.gov (W.J.C.); aaron.schneiderman@va.gov (A.I.S.); 2 San Francisco VA Health Care System, San Francisco, CA 94121, USA; shira.maguen@va.gov; 3 Department of Psychiatry and Behavioral Sciences, School of Medicine, University of California—San Francisco, San Francisco, CA 94143, USA

**Keywords:** post-9/11 veterans, food insecurity, housing instability, social determinants of health, women’s health

## Abstract

Food and/or housing instability (FHI) has been minimally examined in post-9/11 US veterans. A randomly selected nationally representative sample of men and women veterans (n = 38,633) from the post-9/11 US veteran population were mailed invitation letters to complete a survey on health and well-being. Principal component analysis and multivariable logistic regression were used to identify FHI’s key constructs and correlates for 15,166 men and women respondents (9524 men, 5642 women). One-third of veterans reported FHI; it was significantly more likely among women than men (crude odds ratio = 1.31, 95% CI:1.21–1.41) and most prevalent post-service (64.2%). “Mental Health/Stress/Trauma”, “Physical Health”, and “Substance Use” were FHI’s major constructs. In both sexes, significant adjusted associations (*p* < 0.01) were found between FHI and homelessness, depression, adverse childhood experiences, low social support, being enlisted, being non-deployed, living with seriously ill/disabled person(s), and living in dangerous neighborhoods. In men only, posttraumatic stress disorder (adjusted odds ratio (AOR) = 1.37, 95% CI:1.14–1.64), cholesterol level (elevated versus normal, AOR = 0.79, 95% CI:0.67–0.92), hypertension (AOR = 1.25, 95% CI:1.07–1.47), and illegal/street drug use (AOR = 1.28, 95% CI:1.10–1.49) were significant (*p* < 0.01). In women only, morbid obesity (AOR = 1.90, 95%CI:1.05–3.42) and diabetes (AOR = 1.53, 95% CI:1.06–2.20) were significant (*p* < 0.05). Interventions are needed that jointly target adverse food and housing, especially for post-9/11 veteran women and enlisted personnel.

## 1. Introduction

Food insecurity is the limited or inadequate access to food because of insufficient money and other resources for food [1]. Other definitions have been used [2] with many derived from a tested, gold-standard module of 18 questions, the US Department of Agriculture’s (USDA) Household Food Security Survey Module (HFSSM) [3]. Housing instability, also known as housing insecurity, has no standard measure or definition [4] but has been described, for example, as being “cost-burdened” (paying more than 30% of monthly income for housing costs and utilities), living with family members or friends, experiencing frequent moves, or being homeless [4,5,6]. Food and housing play prominently in the lives of Americans with more than 49 million veterans and non-veterans resorting to the use of food banks [7] and nearly 40 million US households reporting that they are cost-burdened [8].

Food insecurity and/or housing instability (henceforth, food/housing instability (FHI)) have been associated in civilian populations with increased morbidity and healthcare use [5,9]. Even though individuals who are experiencing these hardships may share common characteristics, such as having low income or being in ill health, these social determinants of health have rarely been examined together or in relation to one another [5,10,11]. Current social programs may be limited because they have not considered food and housing jointly [10]. Thus, research that addresses these issues together may help in the development of more targeted interventions by improving the identification of those individuals who are most vulnerable [12].

Post-9/11 US veterans, men and women who served during Operation Enduring Freedom/Operation Iraqi Freedom/Operation New Dawn (OEF/OIF/OND) from September 2001 onward, may be at greater risk of food insecurity [13,14,15] and housing instability [6,16,17] than veterans who have served in other conflicts, but this has not been consistently demonstrated. For example, although homelessness has been growing among younger post-9/11 veterans, veterans 55 years of age or older in one study comprised most of the homeless veteran population [18]. In another study, older Vietnam-era veterans and those who served between 1975 and 1990 represented the largest percentages of working-age veterans with food insecurity (12.5% and 12.6%, respectively) relative to post-9/11 (September 2001 or after) (10.7%) and pre-9/11 Gulf War (August 1990–August 2001) veterans (9.5%) [1].

Findings about whether FHI among post-9/11 veterans is greater for women or men has not been adequately addressed. Some research has indicated that the use of federal food assistance programs, for instance, was greater among recent women veterans compared to men [13,15], while other research on food insecurity in post-9/11 veterans showed no difference [19,20]. One recent study showed that working-age female veterans had higher food insecurity (13.5%) than males (10.7%), but these findings were not specific to post-9/11 veterans [1]. Inconsistencies have also been found across studies regarding differences by sex on housing instability among veterans [12] and post-9/11 veterans specifically [17,21,22].

Furthermore, research has not examined FHI in relation to a range of socioeconomic, health, and service characteristics. Characteristics that have generally had singular focus in past investigations of post-9/11 veterans include trauma history [16,22,23], social support [23], and mental health [19,20,24]. Physical health (e.g., disability, obesity, diabetes), household characteristics (e.g., size, number of children), region/urbanicity/rurality, and marital status and other socioeconomic characteristics have been used in analyses among post-9/11 and other veterans [1,16,20,22]. In the general US population, dietary inadequacy was a characteristic of housing unstable families [5,8,10] and renters who were cost-burdened spent 38% less on food [8]. Service characteristics (e.g., military rank, branch, combat exposure, service period) have been minimally addressed among post-9/11 veterans [19,21,22]. Additionally, there has been little study of the association between FHI and gender/sexual orientation in veterans overall [17,25]; Millenium Cohort Study (MCS) data indicated that gay, lesbian, or bisexual veterans had an increased risk of homelessness [17]. Furthermore, there is scarce information about when FHI occurs, such as before, during, or after service. Several studies evaluated data collected only from US Department of Veterans Affairs (VA) healthcare users [20,21,22,25], where findings were not generalizable to the post-9/11 or broader veteran populations.

The current study addresses the very limited investigation of FHI in a nationally representative sample of men and women from the post-9/11 US veteran population. The principal objective was to assess FHI prevalence and occurrence, explore its underlying constructs, and compare its correlates between men and women.

## 2. Materials and Methods

### 2.1. Study Sample and Data Collection

The 2018 Comparative Health Assessment Interview (CHAI) Research Study is a cross-sectional, nationwide survey of the health and well-being of post-9/11 US veterans and non-veterans. Although non-veterans with no military experience (n = 16,843) were included in the study design, this analysis only focused on veterans. The core veteran sample consisted of adults 18 years of age and over with a 30% oversample of women. It was obtained via the stratified random sampling of the VA’s US Veterans Eligibility Trends and Statistics (USVETS) database. Deployed veterans were stratified by military branch (Army, Air Force, Marines, Navy), military component (Active Duty, Reserves/National Guard), and first activation date (i.e., before or after 9/11) and the non-deployed were matched across these strata [26].

Data were collected between April and August 2018. A mixed-mode data collection methodology was administered that entailed completion of a web-based questionnaire or computer-assisted telephone interview. For veterans who were asked to participate (n = 38,633), initial mail contacts were made that included a USD 1 pre-incentive. A USD 50 post-incentive was provided upon survey completion. Reminder calls were made to veterans who did not respond. Informed consent was obtained from all participants prior to survey administration. Study procedures were approved by the VA Central Institutional Review Board.

The veteran response rate was 39.5% (n = 15,170 eligible, returned surveys). Four veterans were removed because deployment status could not be ascertained. The final analytical sample consisted of 15,166 veterans (n = 9524 men; n = 5642 women).

### 2.2. Measures

#### 2.2.1. Outcome Variable

Lifetime FHI was based on two questions that asked about (1) having enough money for food or a place to live and (2) when FHI occurred (“Have you ever not had enough money for food or a place to live?” (yes/no); “Did it happen (SELECT ALL THAT APPLY): 1. Before military service, 2. During military service, 3. After military service”). Questions were adapted from the Life-Stressor Checklist-Revised (LSC-R) [27], a self-report measure that screens for 30 stressful life events (e.g., death, assault) that occur during adulthood or childhood using binary “yes/no” response options. The portion of our survey question on having enough money for food approximated text from the general food sufficiency screener of the 18-item HFSSM, which is part of the broader Current Population Survey’s Food Security Supplement [3]. Our evaluation of food insecurity was limited to resource adequacy and in our analysis was termed “food instability.” Additionally, housing instability has been generally defined more narrowly in past research, namely as whether a veteran has ever been homeless or not. However, our housing instability question, “Having insufficient funds for a place to live”, approaches the topic more broadly without specific reference to homelessness and thus may include other housing issues, such as being cost-burdened while renting/owning a home or while paying rent to friends or family.

#### 2.2.2. Independent Variables

Social support was assessed using the Multidimensional Scale of Perceived Social Support (MSPSS) that was shown to have good reliability [28]. Veterans rated 12 questions on perceived social support from family, friends, and significant others using 7-point Likert scales (1 = very strongly disagree; 2 = strongly disagree; 3 = mildly disagree; 4 = neutral; 5 = mildly agree; 6 = strongly agree; 7 = very strongly agree). Scores were summed across the 12 items with total scores ranging from 12 to 84 (scores less than 12 were set to missing). The higher the MSPSS score, the greater the level of social support. Total scores were evaluated as a three-level ordinal variable: scores between 12 and 35 were classified as low support, those between 36 and 60 as medium, and those greater than 60 as high.

Measures of adverse childhood experiences (ACEs) (e.g., ever lived in dangerous neighborhood, ever lived with seriously ill/disabled person) and potentially traumatic events (PTEs) (e.g., ever bullied, ever sexually assaulted) came from the LSC-R [27] and Life Events Checklist for Diagnostic and Statistical Manual for Mental Disorders-Fifth Edition (LEC-5) [29]. CHAI measurement and content experts selected items from these measures to reduce respondent burden while still covering important content areas [26]. Twenty-four ACE events were evaluated dichotomously (yes/no). The number of events endorsed were summed to generate a total ACE score and converted to a 4-level ordinal variable (“no events”, “1”, “2–3”, “4 or more events”). Likewise, the number of events endorsed out of 22 PTEs was totaled, and a 5-category ordinal variable created (“0”, “1–2”, “3–4”, “5–7”, and “8 or more PTEs”). We also asked about specific life stressors that included whether veterans ever lived in dangerous housing or neighborhoods and if they ever lived with a seriously ill/disabled person.

Lifetime homelessness was assessed utilizing a question from the Vietnam-Era Health Retrospective Observational Study [30]: “Have you ever been homeless for a period of 2 weeks or more at any time in your life? By homeless, we mean times when you did not have a regular place to sleep or the place or places you did sleep were at a shelter, hotel, or somewhere that is not normally used for sleeping (such as a car, park, or train station)”. Respondents answered yes or no. We classified homelessness as a traumatic event [31].

The General Health Scale, obtained from the 12-item Short-Form^TM^ Health Survey (SF-12) [32], was used to examine self-reported health perception (“In general, would you say your health is excellent, very good, good, fair, or poor?”). The SF-12 has comparable validity to the longer, widely used Medical Outcomes Study’s 36-Item Short-Form Health Survey (SF-36) [32]. Scale responses were collapsed into a two-level variable (“excellent/very good/good health” versus “fair/poor health”).

Dietary adequacy was assessed using a question from the Well-Being Inventory (WBI) (“Over the last 3 months, how often have you eaten a generally healthy diet (for example, low fat, limited sugar, adequate servings of fruits and vegetables)?”) [33]. Responses were based on 5-point Likert scales (“never”, “rarely”, “sometimes”, “often”, and “most or all of the time”) and recoded into a two-level variable (“most/all of the time” versus “rarely/never/sometimes”).

Lifetime diabetes, hypertension, hypercholesterolemia, and heart disease were each assessed dichotomously (yes/no) using the 2018 National Health Interview Survey (NHIS) question [34]: “Has a doctor or other healthcare provider ever told you that you had any of the following conditions?”. The number of endorsements to each of these conditions plus 20 additional physical conditions (including obesity) was summed to obtain a total count of physical conditions and analyzed as a four-level ordinal variable (“no conditions”, “1”, “2”’, “3 or more conditions”). Body mass index (BMI), on which “obesity” was based, was calculated from current height and weight and classified as “underweight (<18.5 kg/m^2^)”, “normal weight (18.5–<25 kg/m^2^)”, “overweight (25–<30 kg/m^2^)”, “obesity (30–<40 kg/m^2^)”, and “morbid obesity (≥40 kg/m^2^)”. PTSD and depression were evaluated dichotomously (yes/no) per the NHIS [34].

Lifetime alcohol and lifetime drug use questions were adapted from the National Institute of Health’s National Institute on Drug Abuse’s Alcohol, Smoking, and Substance Involvement Screening Test) (ASSIST) [35]. These substances were analyzed as binary variables (yes/no): cigarettes, cigars, pipes, snuff, or smokeless tobacco; electronic cigarettes (e-cigarettes, vaping); alcohol (including excessive use: four or more drinks/day for women, five or more drinks/day for men); medical marijuana obtained by prescription, or from a medical marijuana dispensary; cannabis (marijuana other than medical marijuana, pot, grass, hash, etc.); prescription drugs for non-medical reasons; illegal or street drugs ever used during the veterans’ lifetime; and the non-medical use of prescription stimulants, sedatives, and opioids, respectively.

Sociodemographic variables included age (18–24, 25–34, 35–44, 45–54, 55–64, 65 or more years), sex (male, female), sexual orientation (heterosexual versus LGBTQ+ (lesbian, gay, bisexual, transgender, queer/questioning, or other gender identity)), race/ethnicity (white, non-Hispanic (single race); black, non-Hispanic (single race); other/multiple races, non-Hispanic; Hispanic (this group includes veterans who identified themselves as Hispanic and of single or multiracial origin)), employment status (paid, full-time/part-time versus unemployed), educational status (high school or less than high school/General Educational Diploma, some college/no degree, associate/technical degree, Bachelor’s degree, graduate degree), marital status (never married, married/domestic partner/civil union, separated/divorced/widowed), household characteristics (e.g., number of children in the household), census region (Northeast, Midwest, South, West), and urban/rural. Urban/rural was defined dichotomously based on the variable “MSA” where “1’” represented a metropolitan statistical area (MSA), defined as an area that contains at least one county with a city population ≥50,000 or a Census Bureau-defined urbanized area with a total population of 100,000 or more [36]; MSA was designated as urban and a non-MSA as rural. The military service variables evaluated were lifetime deployment status (ever deployed, never deployed), military branch (Army, Marine Corps, Air Force, Navy/Coast Guard; based on the CHAI question “In which branch did you serve the most time after September 11, 2001?”), military component (Active, Reserve, National Guard; based on data from USVETS on the last component served), and rank (officer/warrant officer, enlisted; based on USVETS data on rank at time of departure from the service).

### 2.3. Statistical Analysis

Final analysis weights were developed that accounted for CHAI’s complex sampling design and non-response. These were calibrated to population frame totals on sex and service-based characteristics from USVETS for veterans who either served on active duty or were activated/deployed at least once between October 2001–December 2015. A resampling-based variance estimation approach (n = 200 replicate weights) was applied based on the (n − 1) rescaling bootstrap method [37]. Point estimates for subgroups were computed using domain analysis [38]. Alpha values of ≤0.05 were deemed statistically significant. *P*-values were adjusted via the Tukey–Kramer method to reduce Type I error inflation resulting from multiple pairwise comparisons. Analyses were performed using SAS Enterprise Guide (Version 8.2, SAS Institute, Inc., Cary, NC, USA) on a Linux platform. All estimates were weighted except where otherwise indicated.

Counts (unweighted) and other descriptive statistics were calculated. The prevalence of FHI was computed for men and women, respectively, as well as when FHI occurred for each time period (i.e., before/during/after military service) and over all time periods. For all remaining crude and adjusted analyses, FHI was evaluated over all time periods. Sex-stratified, bivariable analysis was performed to examine crude associations between FHI and each of forty-one socioeconomic, health, and military characteristics suggested as correlates by the extant literature. Crude associations were tested via the Rao–Scott design-adjusted chi-square statistic. Principal component analysis (PCA) (unweighted) using orthogonal rotation was applied to reduce the number of potential correlates and identify a set of components that accounted for most of the variance in those correlates [39]. Missing information and variables were removed after formatting the data for input into PCA, which also involved required checks for variable redundancy [39,40], as well as assessing PCA’s adequacy for the data using Bartlett’s test of sphericity and the Kaiser–Meyer–Olkin test [39,41]. PCA solutions that contained components with highly cross-loaded variables (i.e., loading at r ≥ 0.32 on at least two components) or had components that lacked at least three highly loaded variables (r ≥ 0.40) were deemed unacceptable [39,41].

Characteristics highly correlated (r ≥ 0.40) to each component were used to describe that component. Each of the resultant nineteen characteristics were then used in multivariable logistic regression to determine their association with FHI after controlling for all of the remaining characteristics. Prior to regression, multicollinearity was tested by calculating tolerance for each independent variable via linear regression procedures [42]; all tolerance estimates fell above the cut-off of 0.40 [42,43]. Crude (OR) and adjusted odds ratios (AOR) and 95% confidence intervals (CI) between FHI and each correlate were calculated.

## 3. Results

### 3.1. Sample Characteristics

Veterans’ mean age was 38.7 years (SE = 0.04), most were married or in a domestic partnership/civil union (63.4%), male (82.7%), and white non-Hispanic (66.4%), had paid employment (81.6%), had some college (no degree) (29%), and were living in the South (47.7%) and urban areas (83.8%) (Table 1). Most had been enlisted personnel (88.2%), deployed (70.5%), Army veterans (50.7%), and on active duty (66.1%).

### 3.2. Prevalence and Occurrence of FHI

**Figure 1:**
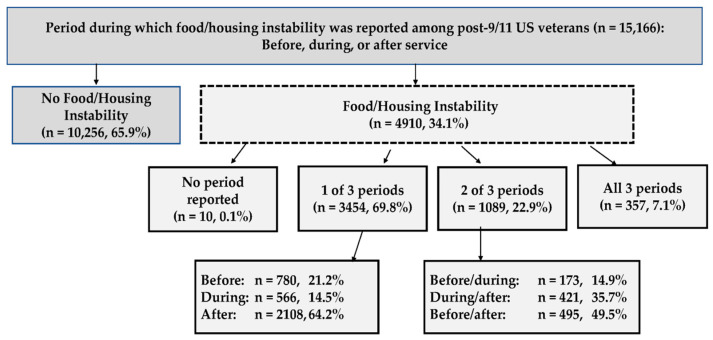
Period(s) during which food/housing instability was reported.

FHI was reported by approximately one-third (34.1%) of post-9/11 veterans (Table 1). Of these, 69.8% reported FHI for one of three periods in their lives (64.2% after service, 14.5% during service, 21.2% before service) (Figure 1). FHI was reported by 22.9% for two of the three periods (49.5%, before/after service; 35.7%, during/after service; 14.9%, before/during service), and by 7% of veterans across all three periods.

### 3.3. Bivariate Unadjusted Results

FHI was significantly (*p* < 0.001) more likely for women (39.2%) than men (33.0%) (crude OR = 1.31, 95% CI:1.21–1.41) (Table 1—footnote ‘b’; Table S1—footnote ‘b’). Characteristics that were more likely among men with FHI than among women with FHI were being deployed, having served active-duty, being heterosexual, being employed, having some college or less, being married or in a domestic partnership/civil union, having moderate/low social support, ever living in dangerous housing or being homeless, being overweight or obese, ever diagnosed with hypertension/high cholesterol/heart condition, having an unhealthy diet, being a cigarette/tobacco user, excessive alcohol use, and lifetime drug use (Table S1).

Characteristics that were more likely among women with FHI than men with FHI were younger age, identifying as LGBTQ+, being a minority, being unemployed, being separated/divorced/widowed/never married, having lived in larger households or in households with children, ever having lived with a seriously ill/disabled person, having experienced eight or more PTEs or four or more ACEs, ever diagnosed with three or more physical health conditions, being morbidly obese, and ever diagnosed with diabetes/depression/PTSD (Table S1).

### 3.4. Principal Component Analysis Results

Table 2 shows variable loadings for the three-component PCA solution. The three components were labeled “Mental Health/Stress/Trauma” (Component 1), “Physical Health” (Component 2), and “Substance Use” (Component 3). The three-component solution explained 43.3% of the cumulative variance in the observed characteristics; components 1 and 2 explained approximately 80% of the variance in the rotated solution (not reported in the table). Nineteen variables had high loadings across the three final PCA components and were deemed correlates of FHI.

Component 1, “Mental Health/Stress/Trauma”, showed stronger positive correlations with “ever been homeless” (r = 0.73), “ever diagnosed with depression” (r = 0.68), “ever lived in dangerous housing/neighborhood” (r = 0.66), “ever diagnosed with PTSD” (r = 0.63), “ever experienced childhood trauma” (r = 0.55), and “ever lived with seriously ill/disabled person” (r = 0.52) (Table 2). “Sexual orientation” (r = −0.40), “perceived social support” (r = −0.48), and “military rank” (r = −0.50) had negative loadings with Component 1. Component 2, “Physical Health”, showed stronger positive correlations with “physical conditions” (r = 0.86), “ever diagnosed with elevated cholesterol” (r = 0.77), “ever diagnosed with hypertension” (r = 0.74), “ever diagnosed with diabetes” (r = 0.69), and “ever diagnosed with heart condition” (r = 0.57). Deployment status (r = 0.44) and BMI (r = 0.41) had lower positive correlations. Component 3, “Substance Use,” showed strong positive correlations with “ever used tobacco products” (r = 0.80), “ever used alcohol in excess” (r = 0.80), and “ever used illegal/street drugs” (r = 0.77).

### 3.5. Multivariable Logistic Regression Results

Table 3 shows unadjusted and adjusted regression results between FHI and each correlate stratified by sex. The estimated odds of FHI among those ever-reporting homelessness were approximately 12 times (*p* < 0.001) the estimated odds of FHI among those who did not report homelessness for men and women, respectively, after adjustment for covariates (men, AOR = 12.24, 95% CI:9.74–15.38; women, AOR = 12.51, 95% CI:9.37–16.68). In both men and women, elevated associations were found between FHI and having lived in dangerous housing/neighborhoods (women, AOR = 3.44, 95% CI:2.87–4.13; men, AOR = 2.68, 95% CI:2.34–3.07) (*p* < 0.001) and having been enlisted (versus officer/warrant officer) (women, AOR = 3.64, 95% CI:2.74–4.83; men, AOR = 2.88, 95% CI:2.33–3.55) (*p* < 0.001). In men and women, respectively, reporting four or more ACEs (versus none) (men, AOR = 2.05, 95% CI:1.60–2.62; women, AOR = 1.65, 95% CI:1.24–2.21) and experiencing low social support (high versus low, men, AOR = 0.56, 95% CI:0.42–0.77; women, AOR = 0.50, 95% CI:0.34–0.73) were significantly associated with FHI (*p* < 0.001). The relationship between FHI and lifetime depression was significant among men (AOR = 1.44, 95% CI:1.22–1.69) (*p* < 0.001) and women (AOR = 1.31, 95% CI:1.07–1.60) (*p* < 0.01), but lifetime PTSD was only significantly (*p* < 0.001) associated with FHI among men (AOR = 1.37, 95% CI:1.14–1.64). No difference was found in the association between FHI and sexual orientation among men and women, respectively, after adjusting for covariates.

The odds of FHI were significantly lower (*p* < 0.001) for the ever deployed than the never deployed among both men and women (men, AOR = 0.67, 95% CI:0.57–0.79; women, AOR = 0.69, 95%CI:0.58–0.83) (Table 3). For women, being morbidly obese (versus normal weight) (AOR = 1.90, 95% CI:1.05–3.42) and lifetime diabetes (AOR = 1.53, 95% CI:1.06–2.20) were significantly associated (*p* < 0.05) with FHI. For men, odds of FHI were significantly lower (*p* ≤ 0.05) for those with high cholesterol versus those with normal cholesterol (AOR = 0.79, 95% CI:0.67–0.92). Odds of FHI in hypertensive men were significantly higher (*p* < 0.05) than for men without hypertension (AOR = 1.25, 95% CI:1.07–1.47). Illegal/street drug use was significantly associated (*p* < 0.01) with FHI in men only (AOR = 1.28, 95% CI:1.10–1.49).

## 4. Discussion

This is the only sex-stratified analysis of adverse food and housing in US post-9/11 veterans. Findings from this analysis were derived from a nationally representative sample and are thus generalizable to the population of post-9/11 veterans. FHI was experienced by a third of these veterans, was found to be more prevalent among women compared to men, and was reported to have mostly occurred after military service. Many of the correlates investigated with respect to FHI were shared by men and women but differences by sex were still evident.

In veteran users of VA healthcare, adjusted associations between “food insecurity” and “homelessness” were of similar magnitude (men, AOR = 13.79, 95% CI: 12.36–15.40; women, AOR = 10.70, 95% CI:9.77–11.70) [44] to estimates reported in our analysis between “food and/or housing instability” and “homelessness” (men, AOR = 12.24, 95% CI: 9.74–15.38; women, AOR = 12.51, 95% CI: 9.37–16.68). It should be noted that lifetime homelessness was not only evaluated with respect to its association with housing instability, which was operationalized more broadly to include situations such as living with relatives or being cost-burdened, but also to its association with food instability; the latter association was demonstrated in an earlier study of US civilian families [10].

Approximately 16–18% of CHAI respondents ever reported being homeless and proportions were found to be similar by sex (16.4% of men versus 17.2% of women, *p* = 0.945) (Table S1, footnote ‘d’). Our estimates were based on a lifetime assessment of homelessness (i.e., “Have you ever been homeless for a period of 2 weeks or more at any time in your life?”) [30] and administered to either users or non-users of VA healthcare. Two prior estimates of homelessness in post-9/11 veterans ranged from approximately 2–4% [17,21]; women veterans, however, were found to be at lower risk of homelessness compared to men [17]. One estimate was based on becoming homeless after separating from the military using data from only users of VA healthcare [21], while the second was derived from MCS prospective data based on post-9/11 era veterans more broadly [17].

Food insecurity in homeless veterans may lead to dietary inadequacies and chronic physical health conditions, such as metabolic-related disorders [45], which was one of the more prevalent groups of ailments among post-9/11 veterans along with musculoskeletal, mental, and nervous system disorders [46]. We also showed significant adjusted, elevated associations between FHI and certain metabolic health conditions, specifically diabetes and morbid obesity in women and hypertension in men. In a recent study of VA healthcare users, food insecurity among veterans showed elevated adjusted odds for men and women, respectively, for diabetes and underweight (compared to normal weight), while the adjusted odds of food insecurity fell below 1.0 for men with hypertension compared to men without hypertension [44]. We found no elevation in the adjusted odds of FHI comparing underweight to normal weight veterans (not reported in tables), and FHI was associated with hypertension in men.

PTSD was independently associated with FHI in men but not in women based on our results. In other research, PTSD was found to be more likely among homeless OEF/OIF men veterans (70.1%) than OEF/OIF women veterans (53.2%) [22]. Elsewhere, this association was stronger for OEF/OIF women versus men (women, hazard ratio (HR) = 1.57, 95% CI:1.09–2.26, *p* < 0.05; men, HR = 1.24, 95% CI: 1.09–1.41, *p* < 0.001) [21]. The adjusted association between food insecurity and PTSD (AOR = 1.05, 95% CI:1.01–1.09) was only found among men VA healthcare users and not women [44]. A greater likelihood of PTSD-related homelessness among post-9/11 men may be associated with a greater propensity for combat involvement among men [22]. Depression was also independently associated with food insecurity among men (AOR = 1.25, 95% CI:1.20–1.31) and women (AOR = 1.22, 95% CI:1.15–1.30) [44] per our findings.

We found that higher numbers of ACEs were associated with FHI. In prior research, ACEs were related to housing instability among other veterans and were the strongest correlates of lifetime housing instability after factors such as age, marital status, race/ethnicity, and mental health disorders [47]. Childhood problems (i.e., abuse, family instability) were reported to be high (40% or more) among homeless veterans, were greater than percentages reported for prior samples of non-homeless veterans, and were related to greater lifetime homelessness and diminished social support [24]. Moreover, homelessness was considered to be a traumatic experience by some and a precursor to mental health issues [31], and in these PCA results, correlations for homelessness were close to those found for other adverse and potentially traumatic experiences, PTSD, and depression. In a novel study, path analysis was used to outline causal associations between ACEs and homelessness based on social support and other mediator variables [23]. The greater application of path analysis, as well as other multivariate techniques, such as the PCA used here, may be useful in gaining a better understanding of the complex, multifaceted relationships that underlie FHI.

Substance use correlates were important, especially for men in our study and in other studies on food and housing issues. In one study, the proportion of homeless post-9/11 men veterans who had substance use disorder (SUD) was far greater (alcohol abuse/dependency, 31.6%; drug abuse/dependency, 25.2%) than that reported for women (alcohol abuse/dependency, 19.2%; drug abuse/dependency, 18.2%) [22]. In another study, adjusted associations between substance use and housing instability were nearly 1.5 times greater among post-9/11 men (AOR = 2.59, 95% CI:2.33–2.87) than women (AOR = 1.85, 95% CI:1.28–2.67) [21]. Additionally, any SUD was significantly associated (*p* = 0.016) with lifetime history of homelessness in post-9/11 men veterans (but not in women) after controlling for other independent variables [23] that parallels findings in our analysis among men. Associations between food instability with tobacco/cigarette use and binge drinking [20] were also reported in post-9/11 veterans, but food instability and substance abuse were not associated among women veteran VA healthcare users [48].

Women with FHI were significantly (*p* < 0.001) more likely to live with children than women without FHI (Table S1). We did not find that this was the case for men, and it was no longer significant for women after adjustment. However, when considering the socioeconomic characteristics of women versus men using CHAI data (e.g., women were more likely to be younger, racially/ethnically diverse, unemployed than men), the challenges faced by recent women veterans became more apparent. According to another source, approximately 30% of veteran women live with children (versus 15.3% of men) and 9.4% live in poverty (versus 6.4% of men) and are more likely to be divorced/separated (22.8%) than men (15.2%) [49]. Veteran households with children and households headed by single women were more likely to be food insecure (26.4% and 23.9%, respectively) than single-male-headed households with children (14.6%) [1]. In our findings, enlisted women, who as enlisted personnel are least compensated for their time in military service, had nearly four times the adjusted odds of FHI than officers, while the odds for enlisted men were 2.9 times the odds for men officers. This disparity was evidenced despite women veterans’ higher level of education compared to men per our findings (48.1% of women earned a Bachelor’s degree or higher versus 39.7% of men), which has also been demonstrated in other studies [15,49]. Post-9/11 veterans with the lowest pay grades comprised the greatest percentage of homeless persons (72.4%) [21], while lower military grade was a risk factor for food insecurity [20]. Paygrade has been viewed as an indicator for post-service employment and socioeconomic status among post-9/11 veterans [17].

Deployed veterans have been found to be at greater risk of some physical and mental health issues compared to non-deployed veterans [50,51,52]. One would suspect that combat-incurred health issues experienced after service may lead to a greater risk of economic instability, poorer social functioning, and possibly FHI. We found, however, that the odds of FHI among ever deployed veterans were lower than the odds for never deployed veterans after controlling for other explanatory variables. One early study revealed that there was no significant association (*p* = 0.29) between food insecurity and the number of deployments in post-9/11 veterans [20]. Another study found that men and women post-9/11 veterans who were homeless were significantly more likely (*p* < 0.01) to report combat-related PTSD than non-combat-related PTSD [22]. Military service overall may be associated with a greater likelihood of food or housing issues because veterans tend to have poorer physical and mental health than non-veterans [53]. This may be related to premilitary factors, such as early life domestic and/or economic instability, or post-military factors, such as service-incurred mental and physical conditions. These issues may hinder their ability to function and integrate fully into their families, community, and society at large. To date, the distinctions among military service, deployment, success of transition, and other service characteristics have not been adequately investigated with respect to FHI and thus require further research.

### Strengths and Limitations

There are strengths and limitations to this analysis. Nationwide, representative data were collected in the CHAI study, the results from which are generalizable to the population of post-9/11 US veterans. The analysis followed survey-design based procedures that to date have been infrequently applied in other studies [54]. Our analysis addressed the shortcomings of the literature by jointly evaluating adverse food and housing in veterans, presenting sex-stratified results, and by examining FHI in relation to a range of other under-examined characteristics using multivariate statistical methods. Moreover, the correlates that were identified as relevant in our research had been cited in other research on food and/or housing in veterans [12,14,47,48,55,56,57].

The CHAI study was based on a retrospective, cross-sectional design and so statements about causality were not possible. Also related to the limitations of cross-sectional designs is the ambiguity that exists about temporality, for example, whether any of the variables presented in this manuscript preceded ever experiencing FHI or whether FHI was antecedent. However, it should be noted that the purpose of our study was not causal or predictive but descriptive in nature, as we were interested in identifying associations between FHI and a range of characteristics. Notably, we found that FHI was more likely to be reported after service, which suggests that it may mostly represent a culmination of factors experienced prior to FHI.

Moreover, self-reported data were subject to recall bias and other measurement errors. Adverse food/housing experiences may be underestimated [58] especially when estimation occurs over an individual’s lifetime [59] as was the case with CHAI. Recall periods may need to be tailored to the specific issues under study to minimize threats to internal validity [60]. Food insecurity was not evaluated using the USDA’s HFSSM and thus important topics on the severity of food insecurity or hunger were not collected or evaluated. Moreover, the FHI question was double-barreled and whether the veteran’s response was based on food and/or housing was not ascertained. Although no widely tested wording for housing instability was administered, it should be noted that there has been no consensus reached about how to uniformly assess or define housing instability [61].

Correlates were derived from PCA, and unlike factor analysis, were obtained without regard to an underlying latent variable structure [62] and generated based on a subjective selection of analytical thresholds. Orthogonal rotation, which produces uncorrelated components, may be limited because it may lose information about the inherent associations that underly social science data [62].

## 5. Conclusions

This study provides a broad analysis of the many characteristics associated with both food and/or housing instability in post-9/11 US veterans. This report showed that certain subgroups of these veterans are particularly vulnerable to FHI, namely women and enlisted personnel. These findings also demonstrate that FHI is not unidimensional but that food and housing instability represent varied socioeconomic issues that require greater multidisciplinary research and interventions. Although too little has been done to alleviate these challenges for veterans, important advances have been made by the VA to attempt to meet those challenges. For example, VA is a contributor to the White House’s National Strategy to end hunger [7,63]. VA is an active participant in this effort by establishing an office of food security, providing information about SNAP to eligible veterans identified via screening, and by establishing collaborations with non-profit organizations to increase veterans’ access to food pantries and other food-related support. VA has also acted to address housing instability by offering emergency housing and permanent housing services to veterans [7]. But the issue still remains about how services for these fundamental needs can be better coordinated and integrated to make outreach and support seamless. This could be more successfully achieved with the continued surveillance of veterans regarding these specific social determinants of health and with the conduct of far more research than presently exists on the joint effects of food and housing instability on veteran health and well-being.

## Figures and Tables

**Table 1:** Characteristics of post-9/11 US veterans, stratified by sex.

	All		Men (n = 9524, 82.7% ^a^)		Women (n = 5642, 17.3% ^a^)		
Characteristic	n	% ^a^	n	% ^a^	n	% ^a^	*p*
FHI ^b^							**<0.001**
Yes	4910	34.1	2893	33.0	2017	39.2	
No	10,256	65.9	6631	67.0	3625	60.8	
Age group (years)							**<0.001**
18–24	295	3.3	186	3.1	109	4.2	
25–34	4487	38.0	2657	37.3	1830	41.3	
35–44	4871	32.5	2835	32.1	2036	34.3	
45–54	3162	16.3	2132	17.0	1030	13.4	
55–64	1893	8.3	1353	8.7	540	6.1	
65 or more	458	1.6	361	1.8	97	0.8	
Mean (SE)	38.7	(0.04)	39.0	(0.05)	37.2	(0.09)	**<0.001**
Sexual orientation							**<0.001**
Heterosexual	14,256	95.6	9250	97.2	5006	88.0	
LGBTQ+	842	4.4	232	2.8	610	12.0	
Marital status, current							**<0.001**
Never married	2475	19.9	1446	19.7	1029	21.0	
Married/domestic	9777	63.4	6619	65.3	3158	54.4	
partner/civil union							
Separated/divorced/	2877	16.7	1433	15.1	1444	24.6	
widowed							
Race/ethnicity							**<0.001**
White, NH	9718	66.4	6568	68.7	3150	55.5	
Black, NH	2344	12.7	1120	10.9	1224	21.0	
Other/multiple races, NH	1397	9.6	869	9.5	528	9.7	
Hispanic	1707	11.4	967	10.9	740	13.8	
Employment, current							**<0.001**
Yes	11,869	81.6	7809	83.4	4060	72.7	
No	3218	18.4	1663	16.6	1555	27.3	
Education, current							**<0.001**
≤HS, GED	1491	12.5	1183	13.7	308	7.2	
Some college/no degree	3913	29.0	2686	29.9	1227	24.8	
Associate/technical degree	2648	17.3	1561	16.7	1087	20.0	
Bachelor’s degree	3951	24.4	2296	23.8	1655	27.3	
Graduate degree	3163	16.8	1798	15.9	1365	20.8	
Region							**<0.001**
Northeast	1367	9.7	887	9.9	480	8.7	
Midwest	2765	20.0	1858	20.5	907	17.3	
South	7602	47.7	4539	46.4	3063	54.0	
West	3360	22.6	2202	23.2	1158	20.0	
Urban/rural							**0.009**
Urban	12,790	83.8	7955	83.5	4835	85.3	
Rural	2328	16.2	1546	16.5	782	14.7	
Deployment status							**<0.001**
Ever deployed	10,868	70.5	7276	74.3	3592	52.5	
Never deployed	4298	29.5	2248	25.7	2050	47.5	
Military branch							**<0.001**
Army	7694	50.7	4857	50.2	2837	52.7	
Marine Corps	1429	13.4	1194	15.1	235	5.7	
Air Force	3338	18.3	1905	17.3	1433	22.8	
Navy/Coast Guard ^c^	2654	17.6	1532	17.4	1122	18.8	
Military component							**<0.001**
Active	9133	66.1	5814	66.7	3319	63.2	
National Guard	3106	18.1	2103	18.2	1003	17.5	
Reserve	2927	15.8	1607	15.0	1320	19.3	
Military rank							0.774
Officer/warrant officer	2175	11.8	1377	11.8	798	11.7	
Enlisted	12,990	88.2	8146	88.2	4844	88.3	

FHI, food and/or housing instability; GED, General Educational Diploma; HS, high school; LGBTQ+, lesbian, gay, bisexual, transgender, queer/questioning, or other gender identity; NH, non-Hispanic; SE, standard error. ^a^ Statistics were weighted (except for counts). Boldface values are statistically significant (*p* ≤ 0.01). ^b^ Crude odds ratio between FHI and sex (women/men, OR = 1.31, 95% CI:1.21–1.41, *p* < 0.001). ^c^ Includes Coast Guard veterans (n = 14).

**Table 2:** Principal component analysis results.

Characteristics	Component 1, “Mental Health/Stress/Trauma”	Component 2, “Physical Health”	Component 3, “Substance Use”
Ever been homeless	**0.73**	−0.07	0.12
Ever diagnosed with depression	**0.68**	0.17	0.00
Ever lived in dangerous housing/neighborhoods	**0.66**	0.00	0.16
Ever diagnosed with PTSD	**0.63**	0.26	0.04
Adverse childhood experiences	**0.55**	0.03	0.23
Ever lived with seriously ill/disabled person	**0.52**	0.07	0.06
Sexual orientation	**−0.40**	0.20	−0.08
Perceived social support	**−0.48**	−0.08	0.04
Military rank	**−0.50**	0.19	−0.15
Physical health conditions	0.27	**0.86**	0.04
Ever diagnosed with high cholesterol	−0.04	**0.77**	0.05
Ever diagnosed with hypertension	0.06	**0.74**	0.05
Ever diagnosed with diabetes	0.06	**0.69**	−0.16
Ever diagnosed with heart condition	0.12	**0.57**	−0.04
Deployment status	−0.15	**0.44**	0.21
Body mass index	0.10	**0.41**	0.02
Ever used tobacco products	0.09	0.06	**0.80**
Ever used alcohol in excess	0.08	0.05	**0.80**
Ever used illegal/street drugs	0.24	−0.05	**0.77**

PTSD, posttraumatic stress disorder. Boldface represents characteristics that loaded high (r ≥ 0.40) on a component. Statistics were unweighted. The variance explained by Components 1, 2, and 3 was 4.30, 4.21, and 2.33, respectively.

**Table 3:** Associations between FHI and correlates, by sex and PCA-derived components.

	Men (n = 9327)		Women (n = 5536)	
Component	Unadjusted OR (95% CI)	Adjusted OR (95% CI)	Unadjusted OR (95% CI)	Adjusted OR (95% CI)
“Mental health/Stress/Trauma”				
Ever been homeless	25.00 (20.41–30.30) ***	12.24 (9.74–15.38) ***	22.73 (17.86–29.41) ***	12.51 (9.37–16.68) ***
*No (Ref)*	−	−	−	−
Ever diagnosed with				
with depression	2.87 (2.57–3.21) ***	1.44 (1.22–1.69) ***	2.37 (2.07–2.72) ***	1.31 (1.07–1.60) **
*No (Ref)*	−	−	−	−
Ever lived in				
dangerous housing/				
neighborhood	6.10 (5.46–6.76) ***	2.68 (2.34–3.07) ***	6.76 (5.88–7.75) ***	3.44 (2.87–4.13) ***
*No (Ref)*	−	−	−	−
Ever diagnosed with				
with PTSD	2.48 (2.20–2.79) ***	1.37 (1.14–1.64) ***	2.29 (1.99–2.62) ***	1.10 (0.90–1.34)
*No (Ref)*	−	−	−	−
Adverse childhood				
experiences				
Four or more	4.92 (4.08–5.92) ***	2.05 (1.60–2.62) ***	4.58 (3.65–5.76) ***	1.65 (1.24–2.21) ***
Two–Three	2.01 (1.64–2.46) ***	1.49 (1.16–1.91) ***	1.81 (1.40–2.35) ***	1.20 (0.86–1.66)
One	1.44 (1.14–1.81) ***	1.23 (0.95–1.59)	1.36 (1.00–1.85)	1.08 (0.76–1.52)
*None (Ref)*	−	−	−	−
Ever lived with				
seriously ill/disabled				
person	3.07 (2.70–3.48) ***	1.53 (1.30–1.81) ***	2.99 (2.58–3.45) ***	1.52 (1.24–1.85) ***
*No (Ref)*	−	−	−	−
LGBTQ+	2.07 (1.51–2.82) ***	1.10 (0.74–1.66)	1.87 (1.52–2.28) ***	1.09 (0.84–1.41)
*Heterosexual (Ref)*	−	−	−	−
Social support				
High score	0.28 (0.22–0.35) ***	0.56 (0.42–0.77) ***	0.30 (0.22–0.41) ***	0.50 (0.34–0.73) ***
Mid score	0.79 (0.62–1.00) *	1.01 (0.74–1.39)	0.67 (0.48–0.93) *	0.79 (0.52–1.19)
*Low score (Ref)*	−	−	−	−
Enlisted	5.45 (4.51–6.59) ***	2.88 (2.33–3.55) ***	6.18 (4.80–7.96) ***	3.64 (2.74–4.83) ***
*Officer/Warrant Officer (Ref)*	−	−	−	−
“Physical Health”				
Physical condition,				
number				
Three or more	1.74 (1.38–2.19) ***	1.27 (0.89–1.80)	2.54 (1.78–3.63) ***	1.33 (0.87–2.04)
Two	1.21 (0.91–1.60)	0.98 (0.68–1.43)	1.71 (1.12–2.62) **	1.15 (0.69–1.91)
One	1.14 (0.87–1.49)	1.18 (0.85–1.65)	1.52 (1.00–2.32) *	1.08 (0.64–1.80)
*None (Ref)*	−	−	−	−
Body mass index (kg/m^2^)				
Obese (30.0–<40.0)	0.94 (0.77–1.14)	0.91 (0.69–1.20)	1.55 (1.24–1.93) ***	1.20 (0.88–1.63)
Morbidly obese (≥40.0)	1.46 (1.02–2.07) *	1.13 (0.71–1.80)	2.25 (1.41–3.56) ***	1.90 (1.05–3.42) *
*Normal weight ^a^ (Ref)*	−	−	−	−
Ever diagnosed with				
high cholesterol	0.88 (0.79–0.98) *	0.79 (0.67–0.92) **	1.13 (0.98–1.30)	0.97 (0.80–1.18)
*No (Ref)*	−	−	−	−
Ever diagnosed with				
hypertension	1.33 (1.20–1.46) ***	1.25 (1.07–1.47) **	1.14 (0.97–1.33)	0.88 (0.70–1.11)
*No (Ref)*	−	−	−	−
Ever diagnosed with				
diabetes	0.93 (0.77–1.12)	0.79 (0.61–1.04)	1.69 (1.29–2.21) ***	1.53 (1.06–2.20) *
*No (Ref)*	−	−	−	−
Ever diagnosed with heart				
heart condition	1.27 (1.09–1.49) **	1.00 (0.79–1.27)	1.23 (0.93–1.61)	0.91 (0.63–1.30)
*No (Ref)*	−	−	−	−
Ever deployed				
*Never deployed (Ref)*	0.63 (0.57–0.71) ***	0.67 (0.57–0.79) ***	0.66 (0.58–0.75) ***	0.69 (0.58–0.83) ***
“Substance Use”				
Ever used alcohol in excess	1.43 (1.30–1.58) ***	1.02 (0.89–1.16)	1.46 (1.29–1.66) ***	1.09 (0.91–1.31)
*No (Ref)*	−	−	−	−
Ever used tobacco products	1.53 (1.36–1.73) ***	1.07 (0.92–1.26)	1.66 (1.46–1.89) ***	1.02 (0.84–1.24)
*No (Ref)*	−	−	−	−
Ever used illegal/street drugs	2.19 (1.97–2.43) ***	1.28 (1.10–1.49) **	1.98 (1.69–2.32) ***	1.19 (0.96–1.47)
*No (Ref)*	−	−	−	−

CI, confidence interval; FHI, food and/or housing instability; LGBTQ+, lesbian, gay, bisexual, transgender, queer/questioning, or other gender identity; OR, odds ratio; PCA, principal component analysis; PTSD, posttraumatic stress disorder; Ref, referent category. Statistics were weighted (except for counts). Boldface values are statistically significant (*p* ≤ 0.05). * *p* < 0.05; ** *p* < 0.01; *** *p* < 0.001. ^a^ Normal weight (18.5–<25 kg/m^2^).

## Data Availability

The VA supports efforts to provide limited, restricted access to research data under written agreements consistent with commitments to protecting subjects’ privacy and confidentiality.

## Supplementary Materials

The following supporting information can be downloaded at: https://www.mdpi.com/article/10.3390/ijerph21030356/s1, Table S1: Prevalence of FHI and bivariable associations with socioeconomic, health, and military characteristics, stratified by sex.
