# Supplementary material for: Prevalence and Correlates of Food and/or Housing Instability among Men and Women Post-9/11 US Veterans
Source: Int J Environ Res Public Health. 2024 Mar 18;21(3):356. doi: 10.3390/ijerph21030356 (PMC10970277; doi:10.3390/ijerph21030356)
Supplement: Supplementary file 1 [file ijerph-21-00356-s001.zip › ijerph-2890637-supplementary.pdf]

Table S1. Prevalence of FHI and bivariable associations with socioeconomic, health, and military characteristics, stratified by sex[a]

	Men (n=9,524, 82.7%[a])					Women (n=5,642, 17.3%[a])				
	Yes, FHI[b] (n=2,893, 33.0%[a])		No, FHI (n=6,631, 67.0%[a])		p	Yes, FHI[b] (n=2,017, 39.2%[a])		No, FHI (n=3,625, 60.8%[a])		p
Characteristic	n	%	n	%		n	%	n	%	
Deployment status					**< 0.001**					**< 0.001**
Ever deployed	2,066	68.3	5,210	77.3		1,208	46.1	2,384	56.6	
Never deployed	827	31.7	1,421	22.7		809	53.9 [c]	1,241	43.4	
Military branch					**< 0.001**					**< 0.001**
Army	1,661	56.2	3,196	47.3		1,111	57.9	1,726	49.3	
Marine Corps	391	15.3	803	15.0		98	6.1	137	5.4	
Navy/Coast Guard	465	17.5	1,067	17.3		414	18.4	708	19.1	
Air Force	361	11.0	1,544	20.4		388	17.6	1,045	26.2	
Military component					**< 0.001**					**0.013**
Active duty	1,842	70.5	3,972	64.8		1,239	65.6	2,080	61.7	
National Guard	610	16.4	1,493	19.2		357	17.6	646	17.4	
Reserve	441	13.1	1,166	16.0		421	16.9	899	20.8	
Rank					**< 0.001**					**< 0.001**
Officer/warrant officer	137	3.4	1,240	16.0		93	3.2	705	17.1	
Enlisted	2,755	96.6	5,391	84.0		1,924	96.8	2,920	82.9	
Age group (years)					**< 0.001**					**< 0.001**
18-24	59	3.0	127	3.2		47	4.6	62	3.9	
25-34	1,017	43.9	1,640	34.0		801	48.2	1,029	36.8	
35-44	1,011	35.5	1,824	30.4		762	34.2	1,274	34.3	
45-54	511	12.1	1,621	19.4		275	9.5	755	15.9	
55-64	253	4.9	1,100	10.6		121	3.2	419	7.9	
65 or more	42	0.6	319	2.4		11	0.2	86	1.2	
Sexual orientation					**< 0.001**					**< 0.001**
Heterosexual	2,765	95.7	6,485	97.9		1709	83.9	3,297	90.6	
LGBTQ+	116	4.3	116	2.1		301	16.1	309	9.4	
Race/ethnicity					**< 0.001**					**< 0.001**
White, NH	1,744	61.1	4,824	72.4		970	49.6	2,180	59.3	
Black, NH	469	15.1	651	8.9		529	25.0	695	18.5	
Other/multiple race(s), NH	335	11.7	534	8.5		208	9.8	320	9.6	
Hispanic	345	12.1	622	10.3		310	15.6	430	12.6	
Employment, current					**< 0.001**					**0.001**
Yes	2,184	75.9	5,625	87.1		1,399	70.1	2,661	74.3	
No	694	24.1	969	12.9		610	29.9	945	25.7	
Education, current					**< 0.001**					**< 0.001**
≤ HS, GED	488	18.2	695	11.4		158	10.0	150	5.3	
Some college/ no degree	1,080	38.8	1,606	25.6		592	32.2	635	19.9	
Associate/technical degree	531	18.3	1,030	15.9		465	23.6	622	17.7	
Bachelor's degree	516	16.8	1,780	27.2		511	22.5	1,144	30.3	
Graduate degree	278	7.9	1,520	19.9		291	11.7	1,074	26.7	
Marital status, current					**< 0.001**					**< 0.001**
Never married	563	23.3	883	17.9		463	25.5	566	18.1	
Married/domestic	1,700	55.0	4,919	70.3		915	45.0	2,243	60.5	

partner/civil union										
Separated/divorced/ widowed	620	21.7	813	11.8		636	29.5	808	21.3	
Household size, current					**0.035**					**0.002**
1	392	13.5	764	11.8		245	10.6	496	12.3	
2	722	24.9	1,980	27.3		493	23.2	1,036	27.0	
3 or more	1,758	61.7	3,854	60.8		1,272	66.1	2,080	60.7	
Household with children, current					0.889					**< 0.001**
Yes	1,435	50.8[c]	3,106	50.7[c]		1,243	65.4	1,932	57.4	
No	1,437	49.2	3,488	49.3		765	34.6	1,680	42.6	
Census region					**0.012**					0.509
Northeast	247	8.7	640	10.5		164	8.4	316	8.9	
Midwest	542	19.5	1,316	21.1		301	17.0	606	17.6	
South	1,417	48.6	3,122	45.3		1,151	55.5	1,912	53.0	
West	676	23.2	1,526	23.1		390	19.1	768	20.6	
Urban/rural					0.157					**0.030**
Urban	2,388	82.6	5,567	84.0		1,701	83.5	3,134	86.4	
Rural	497	17.4	1,049	16.0		306	16.5	476	13.6	
Perceived social support (MSPSS)					**< 0.001**					**< 0.001**
High score	1,404	48.1	4,844	73.3		1,053	53.2	2,633	73.3	
Middle score	1,119	40.6	1,405	21.9		724	35.8	800	22.2	
Low score	328	11.3	315	4.8		223	11.0	170	4.6	
Ever lived with person with serious illness/ disability					**< 0.001**					**< 0.001**
Yes	847	29.0	811	11.8		661	34.0	548	14.7	
No	2,046	71.0	5,820	88.2		1,356	66.0	3,077	85.3	
Ever homeless[d]					**< 0.001**					**< 0.001**
Yes	1,215	43.6	175	3.0		772	39.6	93	2.8	
No	1,678	56.4	6,456	97.0		1,245	60.4	3,532	97.2	
Ever lived in dangerous housing/ neighborhood					**< 0.001**					**< 0.001**
Yes	1,573	55.1	1,095	16.8		1,024	51.8	492	13.7	
No	1,320	44.9	5,536	83.2		993	48.2	3,133	86.3	
Potentially traumatic events, lifetime					**< 0.001**					**< 0.001**
0 events	85	3.4	722	12.3		47	2.9	342	11.2	
1-2	296	10.8	1,522	24.0		164	9.2	805	23.4	
3-4	431	15.8	1,508	22.2		293	15.7	847	23.2	
5-7	811	28.5	1,697	24.3		546	27.3	961	25.8	
8 or more	1,270	41.7	1,182	17.3		967	45.0	670	16.3	
Adverse childhood events					**< 0.001**					**< 0.001**
No events	515	17.9	2,494	37.0		369	17.3	1,283	35.2	
1	428	13.9	1,337	20.0		264	13.1	732	19.7	
2-3	675	23.2	1,560	23.9		429	20.6	828	23.2	
4 or more	1,275	45.1	1,240	19.0		955	49.1	782	21.9	
General health perception, current					**< 0.001**					**< 0.001**

Excellent/very good/ good	1,840	64.2	5,459	82.6		1,316	67.0	2,966	82.3	
Fair/poor	1,049	35.8	1,167	17.4		700	33.0	653	17.7	
Physical health conditions, lifetime					**< 0.001**					**< 0.001**
No conditions	188	7.8	621	11.4		105	6.3	364	12.9	
1	331	13.2	952	17.1		208	11.9	499	16.0	
2	327	12.3	942	15.0		236	13.2	544	15.8	
3 or more	2,047	66.7	4,116	56.5		1,468	68.5	2,218	55.3	
Body mass index (kg/m^2)					**< 0.001**					**< 0.001**
< 18.5	17	0.7	22	0.3		26	1.4	35	1.0	
18.5 – < 25	495	19.2	1014	16.9		499	28.0	1,150	33.1	
25.0 – < 30	1,049	36.3	2,850	43.1		620	29.6	1,290	35.8	
30.0 – < 40	1,169	39.0	2,499	36.7		747	35.7	1,002	27.3	
≥ 40	140	4.8	196	2.9		113	5.3	107	2.8	
Hypertension, lifetime[e]					**< 0.001**					0.102
Yes	1,087	33.8	2,158	27.8		454	19.8	747	17.8	
No	1,806	66.2	4,473	72.2		1,563	80.2	2,878	82.2	
Diabetes, lifetime[e]					0.445					**< 0.001**
Yes	204	4.8	483	5.2		126	5.0	138	3.0	
No	2,689	95.2	6,148	94.8		1,891	95.0	3,487	97.0	
High cholesterol, lifetime[e]					**0.017**					**0.099**
Yes	939	26.8	2,316	29.4		483	20.5	822	18.6	
No	1,954	73.2	4,315	70.6		1,534	79.5	2,803	81.4	
Heart condition, lifetime[e]					**0.004**					0.152
Yes	277	7.8	542	6.2		141	6.5	212	5.4	
No	2,616	92.2	6,089	93.8		1,876	93.5	3,413	94.6	
Had healthy diet, past 3 mos.					**< 0.001**					**< 0.001**
Most/all of the time	1,323	44.8	3,930	58.4		1,067	52.3	2,393	65.6	
Rarely/never/ some times	1,551	55.2	2,660	41.6		949	47.7	1,220	34.4	
Depression, lifetime					**< 0.001**					**< 0.001**
Yes	1,098	39.1	1,154	18.3		1,134	55.8	1,255	34.8	
No	1,795	60.9	5,477	81.7		883	44.2	2,370	65.2	
PTSD, lifetime					**< 0.001**					**< 0.001**
Yes	975	34.7	1,063	17.7		773	36.2	746	19.9	
No	1,918	65.3	5,568	82.3		1,244	63.8	2,879	80.1	
Cigarette/other tobacco use lifetime					**< 0.001**					**< 0.001**
Yes	2,201	77.2	4,465	68.8		1,203	60.6	1,753	48.0	
No	692	22.8	2,166	31.2		814	39.4	1,872	52.0	
E-cigarettes, lifetime					**< 0.001**					**< 0.001**
Yes	927	35.8	1,125	20.1		516	28.4	517	15.6	
No	1,966	64.2	5,506	79.9		1,501	71.6	3,108	84.4	
Alcohol, lifetime										
Yes	2,715	93.9	6,192	93.5		1,859	91.7	3,267	89.9	
No	178	6.1	439	6.5		158	8.3	358	10.1	

Of those with lifetime alcohol use, ever excessively used					**< 0.001**					**< 0.001**
Yes	1,815	67.3	3,505	58.4		1,019	53.8	1,455	44.4	
No	900	32.7	2,687	41.6		840	46.2	1,812	55.6	
Medical marijuana, lifetime					**< 0.001**					**< 0.001**
Yes	319	12.1	245	4.3		180	9.9	123	3.5	
No	2,574	87.9	6,386	95.7		1,837	90.1	3,502	96.5	
Cannabis (not for medical reasons), lifetime					**< 0.001**					**< 0.001**
Yes	1,050	38.1	1,349	21.5		572	30.0	548	16.1	
No	1,843	61.9	5,282	78.5		1,445	70.0	3,077	83.9	
Use of illegal/street drugs, lifetime					**< 0.001**					**< 0.001**
Yes	988	35.7	1,285	20.2		501	25.8	508	14.9	
No	1,905	64.3	5,346	79.8		1,516	74.2	3,117	85.1	
Ever used prescription drugs for nonmedical reasons					**< 0.001**					**< 0.001**
Yes	441	16.9	385	6.9		229	12.2	169	5.2	
No	2,452	83.1	6,246	93.1		1,788	87.8	3,456	94.8	
Of those who have ever used prescription drugs (for non-medical reasons):										
Ever used prescription stimulants					**0.002**					0.597
Yes	219	52.8[c]	156	41.9		101	45.1	69	42.2	
No	222	47.2	229	58.1		128	54.9	100	57.8	
Ever used prescription sedatives					**0.007**					0.951
Yes	267	60.0	196	50.4		134	59.3	101	59.7	
No	174	40.0	189	49.6		95	40.7	68	40.3	
Ever used prescription opioids					0.412					0.087
Yes	303	69.2	249	66.2		153	68.8	101	60.0	
No	138	30.8	136	33.8		76	31.2	68	40.0	

BMI (kg/m^2), body mass index: < 18.5 (underweight), 18.5 - < 25 (normal weight), 25.0 - < 30 (overweight), 30.0 - < 40 (obese), ≥ 40 (morbidly obese); FHI, food and/or housing instability; GED, General Educational Diploma; HS, high school; LGBTQ+, lesbian, gay, bisexual, transgender, queer/questioning, or other gender identity; MSPSS, MSPSS, Multidimensional Scale of Perceived Social Support; NH, non-Hispanic.

[a] Statistics were weighted (except for counts). Boldface indicates values that are statistically significant ($p \leq 0.05$).

[b] Crude odds ratio (OR) between FHI and sex (Women: men, OR=1.31, 95% confidence interval:1.21-1.41, $p < 0.001$).

[c] Higher percentage found for lower unweighted count reflects the assigned weights which here response-bias adjusts for the non-deployed veterans' lower propensity to respond to the survey.

[d] Ever being homeless was reported for 16.4% of men and 17.2% of women ($p = 0.945$).

[e] Age at diagnosis mean(standard error): diabetes, 41.6(0.40); hypertension, 34.2(0.16); hypercholesterolemia, 36.4(0.16); heart condition(s), 35.4(0.55).
